# Role of CBCT in Prediction of Oro-antral Communication Post Third Molar Extraction: A Retrospective Study

**DOI:** 10.1055/s-0043-1760720

**Published:** 2023-06-19

**Authors:** Anupam Singh, Murali Venkata Rama Mohan Kodali, Kalyana Chakravarthy Pentapati, Anwesha Chattopadhyay, Rhea Shetty, Vathsala Patil, Srikanth Gadicherla, Komal Smriti

**Affiliations:** 1 Department of Oral and Maxillofacial Surgery, Manipal College of Dental Sciences, Manipal, Manipal Academy of Higher Education (MAHE), Manipal, Karnataka, India; 2 Department of Oral and Maxillofacial Surgery, College of Dentistry, King Faisal University, Al-Ahsa, Kingdom of Saudi Arabia; 3 Department of Public Health Dentistry, Manipal College of Dental Sciences, Manipal, Manipal Academy of Higher Education (MAHE), Manipal, Karnataka, India; 4 Department of Prosthodontics & Crown and Bridge, Manipal College of Dental Sciences, Manipal, Manipal Academy of Higher Education (MAHE), Manipal, Karnataka, India; 5 Department of Conservative Dentistry and Endodontics, Manipal College of Dental Sciences, Manipal, Manipal Academy of Higher Education (MAHE), Manipal, Karnataka, India; 6 Department of Oral Medicine and Radiology, Manipal College of Dental Sciences, Manipal, Manipal Academy of Higher Education (MAHE), Manipal, Karnataka, India

**Keywords:** CBCT scan, oro-antral communication, maxillary third molar, tuberosity fracture

## Abstract

**Objective** Oro-antral communication (OAC) is one of the most frequently encountered complications during third molar extraction. Various radiographic factors, like excessive maxillary sinus pneumatization, long periods of edentulism, periapical lesions, etc., have been considered high-risk factors for OAC. However, a panoramic radiograph has not proven to be accurate in predicting the chances of OAC. Through this retrospective study, we evaluated the efficacy of a CBCT in predicting the incidence of OAC after maxillary third molar extraction.

**Materials and Methods** We conducted a retrospective study in our department, which included the patients who had undergone extraction of a maxillary third molar over five years with the presence of panoramic X-rays and/or CBCT scans prior to extraction. Primary outcomes assessed from the case files were intra-operative complications like OAC, root fracture, tuberosity fracture, pterygoid plate fracture, etc. The incidence of these complications was correlated with the presence or absence of CBCT before extraction.

**Results** Out of 920 extracted maxillary third molar, only 148 teeth (16.1%) had a CBCT record before extraction. The most commonly encountered complication was broken inaccessible root piece/s (4.9%), followed by OAC (3.5%). An inter-group comparison showed that a significantly higher percentage of patients (p < 0.001) with CBCT records had an incidence of OAC (11.5%) as against the group of patients with no CBCT record (1.9%).

**Conclusion** A CBCT scan prior to cases with high-risk factors for OAC can be a valuable tool in accurately predicting the chances of OAC after maxillary third molar extraction.

## Introduction

The extraction of maxillary third molars is performed for therapeutic and prophylactic reasons. 1 Even though surgically less challenging than mandibular third molar extraction, the maxillary third molars should be adequately assessed because of their anatomic location. The maxillary sinus floor is highly variable, and its proximity to posterior maxillary teeth remains an area of concern while extracting these teeth. 2 The proximity of the maxillary sinus floor to the apices of posterior maxillary teeth depends on various factors like the age of the patient, period of edentulism, maxillary sinus pneumatization, etc. 3 This proximity of the root apices and maxillary floor poses the risk of oro-antral communication after third molar extraction if there is a thin or negligible layer of bone between them. Any periapical or periodontal infection can further complicate the situation, eventually leading to erosion of the intervening bone layer. 4

Apart from the oro-antral communication (OAC), the difficult access to the maxillary third molar can lead to difficulty retrieving broken root pieces. The dislodgement of the tooth or the broken root pieces to surrounding anatomic areas is another concern during surgical extraction of the maxillary third molar. One of the most feared complications is the displacement of the tooth into the infratemporal fossa, the retrieval of which is a surgical challenge. 5 6

Fracture of maxillary tuberosity during the maxillary third molar extraction is a complication that can cause grave concern if not appropriately addressed. The maxillary tuberosity fracture can alter the maxillary alveolus morphology, making prosthodontic rehabilitation challenging. Divergent roots, hypercementosis, ankylosis, isolated tooth, over-eruption, and enlarged and thinned maxillary sinus walls can fracture maxillary tuberosity. 7 Fracture of maxillary tuberosity can further complicate the situation if it results in tethering or the fracture of posteriorly situated medial and lateral pterygoid plates. Due to its critical anatomical position, fracture of the pterygoid plates can result in severe bleeding post-extraction, which will need intervention by an oral surgery specialist. 8

While complications like OAC are unavoidable, they can be managed effectively by a thorough clinical and radiological examination. Two of the most widely accepted and clinically relevant classification schemes have been Pell and Gregory's spatial orientation classification and Archer's Sinus approximation classification. 9 10 11 A panoramic radiograph has been used as a screening tool to assess the position of the maxillary third molar. However, accurate spatial assessment of a third molar is impossible with a two-dimensional radiograph. Hence, a cone-beam computed tomography (CBCT) scan of the maxillary third molar can help better visualize and analyze its location, angulation, spatial orientation, and relation to the surrounding structures. Various studies have analyzed different risk factors for the complications associated with maxillary third molar extraction. 1 9 12 Recently, some studies have been conducted that have compared the CBCT with panoramic radiographs to assess the relation of the maxillary sinus floor to the maxillary molar teeth apex. 13 14 Iwata et al. (2021) compared the Computed Tomography (CT) scan against panoramic radiography in predicting the incidence of OAC. 15 However, CBCT is considered safer and more cost-effective in evaluating the dentofacial region and is readily available for dental practitioners.

The primary objective of this study was to compare the usefulness of a CBCT against a panoramic radiograph in predicting the OAC. The authors have also proposed an algorithm to help the clinician decide the need for CBCT prior to extraction. In addition, the authors also wanted to assess the incidence of other complications during the third molar extraction.

## Materials and Methods

A retrospective study was carried out, which included the patients who had undergone extraction of a maxillary third molar from October 2016 to October 2021 at the Department of Oral & Maxillofacial Surgery. Before the commencement of the study, ethical approval was obtained from the Institutional Ethics Committee (IEC 1073-2019). The database was searched for maxillary third molar extractions of the adult patients, and the case records, and radiographs, including the Cone-Beam CT scan, were accessed. 1268 patients were found to have undergone the procedure in the period mentioned above. The files were screened manually, and the radiographs like panoramic X-ray and CBCT were screened digitally for each patient who had undergone maxillary 3rd molar extraction. Only the patients with a well-documented case with either panoramic X-ray and/or CBCT were included in the study. After screening, 168 patients were excluded from the study because of the inability to retrieve the data or the unavailability of any radiographs.

The files were screened independently by two researchers (A.B. and R.S.). The researchers were oriented about the methodology of data collection. The data collected included the demographics of the patient, the tooth extracted, and the method of extraction (open or closed). Using the available radiographic evidence, the maxillary third molar angulation was categorized as per Archer's classification and the eruption status as per Pell and Gregory's classification (Class A, B, C). Additionally, the proximity of the maxillary third molar to the maxillary sinus floor was categorized as Sinus Approximation (2mm or less bone available between the root tip and the maxillary sinus floor) or No sinus approximation (more than 2mm bone available in between the root tip and the maxillary sinus floor). The files were also screened for post-extraction complications – oro-antral communication, tuberosity fracture, or inaccessible retained root piece. Any discrepancy between the two observers was resolved by an independent researcher (A.S.)

### Statistical Analysis

All the analysis was done using SPSS version 18. A p-value of <0.05 was considered statistically significant. Categorical variables were compared using the Chi-square test.

## Results

Nine hundred twenty maxillary 3rd molars, comprising 519 females (56.4%) and 401 males (43.6%), were included in the study. Of these teeth, 518 (56.3%) were on the left side, and 402 (43.7%) were on the right side. The age group of the patients ranged from 16 to 67 years old. All the patients had a record of panoramic X-ray, while only 148 teeth (16.1%) had an additional CBCT record before extraction. The closed method extraction was done for seven hundred forty-three teeth (80.8%), while 177 (19.2%) were extracted by the open or trans alveolar method.

### Radiographic Classification

Out of all the teeth extracted, the majority of the teeth had vertical angulation (79.5%), followed by distoangular (13.3%), mesioangular (4.8%), and buccoangular (2.5%). With regards to the depth of impaction, the majority of maxillary 3rd molar extracted belonged to Pell and Gregory Class A type (75.6%), followed by Class B (16.2%) and Class C (8.2%). Based on the criteria given by Pell and Gregory, 39.7% of the maxillary 3rd molars were found to have Sinus Approximation (SA), while 60.3% of the teeth had no Sinus Approximation (NSA).

### Incidence of Complications

The most commonly encountered complication was broken inaccessible root piece/s, noted in 45 patients (4.9%). Oro-antral communication (OAC) was reported in 32 patients (3.5%). Another lesser commonly encountered complication was tuberosity fracture in 19 patients (2.1%) and pterygoid plate fracture in 1 patient. ( Table 1 )

**Table 1:** Incidence of complications after third molar extraction

Incidence	N	%
OAC	32	3.5%
Tuberosity fracture	19	2.1%
Pterygoid plate fracture	1	0.1%
Inaccessible root pieces	45	4.9%

### Relationship with the Presence of CBCT Record and Complications Associated with Maxillary Third Molar Extraction

An inter-group comparison showed that a significantly higher percentage of patients (p < 0.001) with CBCT records had an incidence of OAC (11.5%) as against the group of patients with no CBCT record (1.9%). However, regarding other complications like fractured tuberosity and inaccessible root pieces, no significant difference was noted between the CBCT group and the non-CBCT group. ( Table 2 )

**Table 2:** Relationship with presence of CBCT record and complications associated with Maxillary third molar extraction

		CBCT				P-value
		Absent		Present		
		N	%	N	%	
OAC	Absent	757	98.1%	131	88.5%	< 0.001
	Present	15	1.9%	17	11.5%	
Tuberosity fracture	Absent	754	97.7%	147	99.3%	0.34
	Present	18	2.3%	1	0.7%	
Inaccessible root pieces	Absent	735	95.2%	140	94.6%	0.752
	Present	37	4.8%	8	5.4%	

## Discussion

The post-surgical complications after mandibular third molar extraction have been researched extensively and mainly comprise complications like inferior alveolar nerve paraesthesia and lingual nerve damage. 16 However, during the extraction of the maxillary third molar, the post-surgical complications are relatively lower as there are no major neurovascular bundles in proximity and less dense bone in the posterior maxilla. 17 OAC, broken, inaccessible root pieces, maxillary tuberosity fracture, and pterygoid plate fracture are some of the intra-operative complications that are generally encountered during surgical or non-surgical removal of maxillary third molar.

In the literature, the incidence of OAC has been reported from 2.4% to 18.7% and is considered the most frequently encountered intra-operative complication. 1 11 Root fracture is also a common complication encountered and has been a significant risk factor for OAC upon retrieving those broken fragments. Rothamel et al. reported a significant increase in the risk of OAC (ranging from 12% to 27%) in association with a root fracture. Other lesser commonly encountered complication includes dislodgement of the tooth into the maxillary sinus, maxillary tuberosity fracture, pterygoid plate fracture, and tethering of buccal fat pad. 8 11 18 The findings of our study reflected a similar picture, with broken root pieces being the most common complication (4.9%) and followed by OAC (3.5%).

OAC after third molar extraction, if not identified at the time of occurrence, can lead to long-term morbidity in chronic maxillary sinusitis, persistent pus discharge, and oro-antral fistula. The clinician routinely uses a pre-operative panoramic radiograph to assess the proximity of the maxillary sinus floor and the root apex. However, a panoramic radiograph has proven to be an unreliable tool in accurately predicting the risk of OAC. 1 9 19

In the past decade, CBCT has gained wide popularity in the diagnosis and treatment planning of various aspects of dentistry. CBCT analysis has been used to study the root and root canal morphology and their anomalies. 20 21 22 23 Along with tooth morphology, digital scans can also be used to study the various aspects of the maxillofacial bones, like their density, porosity, associated fracture, pathology, or anatomic relations. 24 25 CBCT has been extensively used to study the axial inclination, spatial orientation, and relation of the mandibular third molar to the inferior alveolar nerve canal. 26 27 28 29 Similarly, CBCT can also be used to evaluate maxillary sinus and help detect any septae or other pathology that can alter the dentoalveolar treatment planning. 30

Jung and Cho suggested that a maxillary sinus floor superimposition on the teeth root on a panoramic radiograph should be further probed to predict the chances of sinus floor perforation. 31 In another study by Jung and Cho, upon CBCT evaluation in most teeth, the sinus floor was on the buccal side of the root. 13 Root projection into the maxillary sinus, presence of periapical infection, discontinuity of maxillary sinus floor, and Sinus approximation are some danger signs on panoramic radiographs regarding the risk of OAC. It has been suggested that in the presence of any of these signs, a CBCT should be advised to evaluate further the relation of the sinus floor to the tooth apex. 14

Iwata et al. (2021) investigated the relevance of CT scans in predicting the incidence of OAC.15 After the initial screening of patients by panoramic radiograph, the patients in high-risk stratification for OAC were further evaluated by CT scan. They concluded that a CT scan was a valuable tool in predicting the OAC and suggested their regular use for better patient counseling and clinician preparation for the management of OAC. However, in the past decade, CBCT has been proven more cost-effective and has lesser radiation exposure with a similar bone definition than a conventional CT scan. 32 33 Hence, we chose to investigate the role of CBCT in predicting OAC, which is more readily available for dental radiographic investigation.

**Fig. 1:**
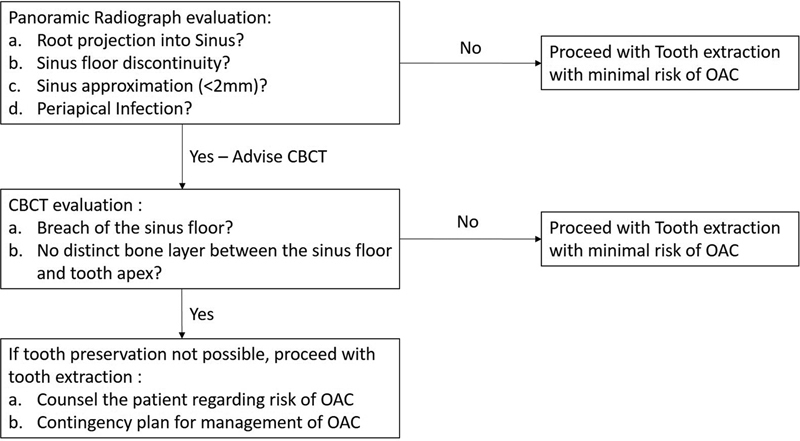
OAC prediction Algorithm post maxillary third molar Extraction

16.1% of patients who underwent maxillary third molar extraction were advised for a CBCT scan in our study. Upon evaluation, it was found that in all these patients, the clinician had found one of the features like root projection into the maxillary sinus, discontinuity of maxillary sinus floor, presence of periapical infection, or sinus approximation (<2mm) on a panoramic radiograph and hence, were advised for CBCT scan. Based on these observations, we have proposed an algorithm to help the clinician make decisions regarding reporting a CBCT scan before the extraction. ( Fig. 1 )

The fact that a significant proportion of patients with CBCT had an incidence of OAC compared to patients with no CBCT shows that the proper evaluation of a panoramic radiograph and identification of risk factors were instrumental in deciding the need for CBCT investigation. CBCT, even though an essential diagnostic tool, can only help the clinician better prepare for an OAC if it happens and also counsel the patient about the possible risk. It is advisable to extract the compromised maxillary third molar if clinically indicated, notwithstanding the risk of OAC. If retained, these compromised teeth can further complicate the condition and, in a worse case, can lead to the spread of infection from the infected molar tooth to the maxillary sinus, leading to maxillary sinusitis.

The apparent drawback of the study is that it is based on a retrospective design. A similar study with a randomized controlled-trial design would better correlate the need for CBCT and identify the risk of OAC. Also, because of the retrospective nature, it was not possible to segregate the patients who had the CBCT records for some other reason. This could have confounded the results and can be better managed in a prospective study design.

## Conclusion

A proper assessment of the available radiographs can help the clinician better prepare for the anticipated complications associated with tooth extraction and also help educate the patient about the same. With the help of our proposed algorithm, we hope to make decision-making more straightforward for the clinician to choose relevant investigations during the third molar extraction.
